# Burden and Costs of Severe Burn Injury in Victoria, Australia

**DOI:** 10.3390/ebj3030034

**Published:** 2022-07-13

**Authors:** Heather Cleland, Ieva Sriubaite, Belinda Gabbe

**Affiliations:** 1 Department of Surgery, Central Clinical School, Monash University, Commercial Rd., Melbourne, VIC 3000, Australia; 2 Victorian Adult Burns Unit, Alfred Hospital, Commercial Rd., Melbourne, VIC 3000, Australia; 3 Centre for Health Economics, Monash Business School, Monash University, 900 Dandenong Rd., Caulfield, VIC 3145, Australia; ieva.sriubaite@monash.edu; 4 School of Public Health and Preventive Medicine, Monash University, 553 St. Kilda Rd., Melbourne, VIC 3000, Australia; belinda.gabbe@monash.edu; 5 Health Data Research UK, Swansea University Medical School, Swansea University, Swansea SA2 8QA, UK

**Keywords:** burns costs, burns burden of injury, activity based funding

## Abstract

This study examines the costs of severe burn injury in Victoria, Australia. It quantifies the funding generated through an activity-based case-mix system for hospital treatment of acute injury and presentations in the subsequent two years and costs of the longer-term burden of burn injury due to premature burn-related deaths and disability. Severe adult burns cases in Victoria from 2007–2016 were identified using the Victorian State Trauma Registry (VSTR). Cases were linked with the Victorian Admitted Episodes Dataset (VAED), Victoria Emergency Minimum Dataset (VEMD), and the National Coronial Information System (NCIS). Hospital re-imbursements and costs of Disability-Adjusted Life Years (DALYs) were calculated using disability weights derived from the EQ-5D-3L questionnaire responses at 24 months post injury. There were 331 patients hospitalised with a burn ≥20% total body surface area (TBSA) from 2007–2016. Total mean re-imbursement (SD) for the acute treatment episode per patient in Australian dollars (AUD) was $87,570 ($97,913). There was significant variation in the number of cases by year and re-imbursement per patient, with high outliers common. Excluding 2009, when 173 people died in bushfires, there were 7749 DALYs which cost $991,872,000. Severe burns are uncommon and variable. Economic treatment costs of severe burns are high, and among survivors there is high incidence of long-term disability and overall burden of injury.

## 1. Introduction

Severe burns are complex injuries with a high risk of mortality and long-term morbidity, and require management in a specialist service [1,2], where they contribute disproportionately and unpredictably to costs [3]. The Australian state of Victoria has one specialist adult burns service based at the Alfred Hospital in Melbourne which treats virtually all Victorian adult patients with severe burns [4]. Hospital funding in the state is largely determined using an activity-based case-mix approach which is designed to provide a ‘rolled-up’ average price for each admission, based on standardised coding [5]. Additional specified grants are allocated for other activities, such as training, and to ‘top-up’ services thought to be underfunded through case-mix. The basis on which such grants are determined, and their purpose, may be opaque. Sub-acute and non-inpatient services are funded through a mix of various methods including block funding and activity-based funding.

Case-mix based methods have been shown to underfund specialist trauma centres for severe injury and underestimate the actual cost of treating burns patients: the ‘true’ or actual costs of burn care are poorly identified [6,7]. Chronic underfunding risks the sustainability of a high-quality specialist service upon which large populations and whole regions depend for regular demand and disaster response.

Therefore, an understanding of the actual financial costs of care, as well as personal and social losses, is required to support the development of a flexible and realistic approach to funding burns care and to ensure the long-term sustainability of a high-quality burns service. As a first step in characterising the costs of burn injury to the community, the aims of this study are to examine the funding generated though case-mix paid to hospitals for burns care and to evaluate the ongoing economic burden of injury for people who have suffered severe burns.

Throughout this paper, the term ‘hospital re-imbursement’ in relation to health care provision refers to funding re-imbursement, or payments, generated through the case-mix system, in contradistinction to ‘cost’, or ‘actual cost’, which refers to the costs incurred by hospitals to treat patients or the calculated burden of injury costs of premature death and disability.

## 2. Materials and Methods

### 2.1. Setting

The Australian state of Victoria had 5,926,624 residents in 2016 [8], nearly 80% of whom resided in the capital city of Melbourne. Victoria operates an inclusive trauma system to ensure that injured patients receive their care at the most appropriate level in the system. Previous research has shown that 98% of adult patients with severe burn injury are managed at the Victorian Adult Burns Service (VABS) housed in the Alfred Hospital in Melbourne [4].

### 2.2. Study Design

All major burns cases (defined as cases involving ≥20% total body surface area) in Victoria were identified using the Victorian State Trauma Registry (VSTR). Cases were linked with the Victorian Admitted Episodes Dataset (VAED), the Victoria Emergency Minimum Dataset (VEMD), and the National Coronial Information System (NCIS). All in-hospital and out-of-hospital deaths due to acute burn injury were identified using the NCIS. Data linkage was performed by the Centre for Victorian Data Linkage (CVDL), based in the Victorian Government Department of Health, and provided for analysis with signed deeds of agreement. Probabilistic linkage methods were used based on identifiers including first name, surname, sex and date of birth. To achieve a higher linkage rate, the CVDL uses “fuzzy matching” on name, and the linkage rate achieved was 98%.

#### 2.2.1. The Victorian State Trauma Registry (VSTR)

The Victorian State Trauma Registry (VSTR) is a state-wide population-based register of all hospitalised major trauma cases in Victoria, used to monitor the performance and effectiveness of the Victorian State Trauma System [9]. All severe burns cases are included in the registry. The registry collects a range of patient characteristics such as age, gender, socio-economic status, and health-related quality of life outcomes determined by EQ-5D-3L questionnaire responses.

#### 2.2.2. Victorian Admitted Episodes Dataset and Victorian Emergency Minimum Dataset

All major burns cases admitted to a hospital in Victoria with a date of injury 1 January 2007 to 31 December 2016 were linked with the VAED and the VEMD. The VAED records demographic, clinical and administrative information for each admitted episode of care at public and private hospitals including rehabilitation centres, extended care facilities and day-procedure centres in Victoria. The VEMD details information for emergency department (ED) presentations at all public hospitals in Victoria.

#### 2.2.3. National Coronial Information System

The NCIS is the database of mortality data on deaths reported to a Coroner in Australia and New Zealand. Deaths that require reporting to the coroner include unexpected deaths and deaths resulting from an accident or trauma, including burns.

### 2.3. Analysis

#### 2.3.1. Inpatient Stay and Emergency Department Re-Imbursement

The Activity Based Funding model introduced in 2011 in Victoria was used to calculate payments to hospitals, including inpatient and emergency presentation. Hospital payments for acute inpatient episodes were calculated using Weighted Inlier Equivalent Separation (WIES) price weights based on Australian National Diagnosis Related Groups (AN-DRGs) multiplied by the price per WIES unit for the relevant financial year. Re-imbursements for inpatient rehabilitation were determined using the subacute WIES (SWIES) cost weight based on the Australian National Subacute and Non-Acute Patient Classification (AN-SNAP) [10]. The reimbursements for patient presentations to the emergency department (ED) were calculated according to the Activity Based Funding model for Emergency Care introduced by the National Health Reform Agreement in 2011. All figures were scaled to 2016 Australian dollars (AUD) using the consumer price index. All dollar amounts are in AUD unless specified otherwise. In the very small number of patients with missing data, the average cost of the care type and nature of injury group for that patient was substituted.

#### 2.3.2. Calculation of Disability-Adjusted Life Years

Disability-Adjusted Life Years (DALYs) were used to quantify the overall burden of injury, comprising the sum of the years of life lost (YLLs) and years lived with a disability (YLDs) [11]. Consistent with previous studies, the disability weight to calculate YLDs was derived from the EQ-5D-3L questionnaire responses recorded in the VSTR [12,13]. The EQ-5D is validated in burns [14]. The EQ-5D-3L includes five items: mobility, self-care, usual activities, pain or discomfort, and anxiety or depression. The respondent is asked to rate their level of problems on each of these items on a three-level scale from no problems to extreme problems or cannot perform The EQ-5D-3L score was assumed to have stabilised by 24 months post injury [15]. Expected life years were defined using the 2013–2015 Australian standard life table [8].

#### 2.3.3. Economic Burden of Severe Burns

The economic burden of health loss (death and disability) was calculated as a cost per DALY, or the value of a year of life free of injury, disease and disability using an adjusted value of statistical life (VLY), as outlined in a guidance note published by the Australian Government in 2020 [16].

## 3. Results

### 3.1. Patient Demographics

From 2007–2016 in Victoria, 597 people died as a result of burn injury. A total of 331 patients aged 16 and over with a burn injury ≥20% TBSA were hospitalised (Table 1). Men comprised 74.3% (246) of admissions. There was no documented pre-existing co-morbidity in 66% of patients, and 83% were less than 65 years old. In total, 74 patients (22.4%) died: more than half (52.5%) of the patients with a burn ≥40% TBSA died during their acute hospital admission. A small proportion of admissions (13.6%) were compensable, funded by private health insurance, workers’ compensation, or transport accident schemes.

### 3.2. Total Re-Imbursements

The median total re-imbursement (IQR) per patient for acute treatment for the study period was $39,343 ($13,844, $137,452), comprising $1403 ($950–$1993) for ED, and $37,256 ($12,398–$135,633) for admissions. The total mean re-imbursement per patient (SD) was $87,570 ($97,913). In the two years following injury, ED presentations and hospital admissions were uncommon. There were 169 presentations to ED or for admission, or both, at a total re-imbursement of $2,003,157. The median re-imbursement per patient of follow-up treatment within two years of injury (ED visits and admissions) was $5120 ($1461, $13,699). The mean total (SD) follow-up re-imbursement per patient was $11,853 ($19,320).

### 3.3. Re-Imbursement by Size of Burn

Re-imbursement for treatment in the ED and for inpatient stays grouped by size of burn are shown in Table 2. Mean re-imbursement increased with size of burn. The highest median re-imbursement of acute hospital admission per patient was for patients with 30–39% TBSA burns, at $93,173 ($26,120, AUD $138,668). For patients with 40–89% TBSA burns, who had a high mortality rate, the median (IQR) re-imbursement was $78,831 ($2729, $214,092) per patient.

### 3.4. Re-Imbursement for Treatment by Year

**Figure 1:**
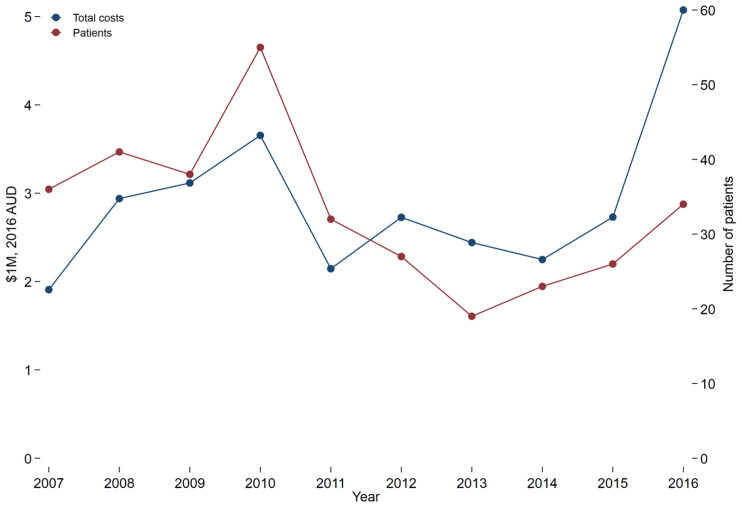
Total acute treatment costs and number of patients by year. Costs in Australian dollars (AUD).

**Figure 2:**
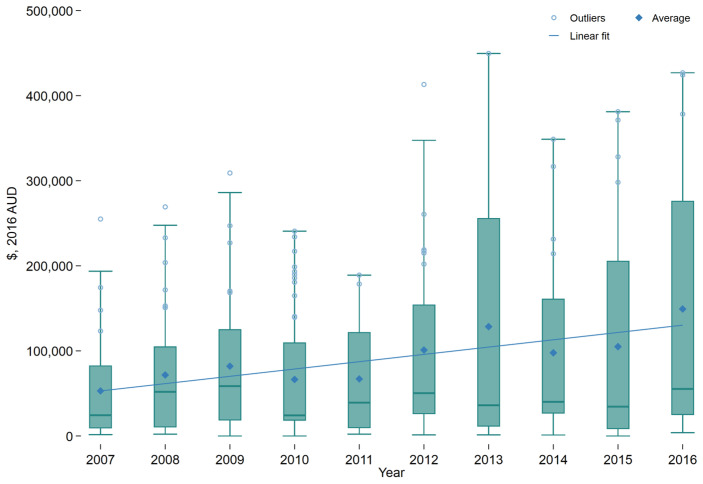
In-patient treatment costs by patient in Australian dollars (AUD) for patients with a cutaneous burn injury by year. The linear fit represents the trend line for the figure. The horizontal line within each box represents the median and the “whiskers” above and below the box represent the 75th and 25th percentiles, respectively.

The number of patients with severe burns who presented to trauma-receiving hospitals each year varied from 19 in 2013 to 55 in 2010. The average number of presentations per year was 33.1. Overall, increased numbers of patients by year were associated with increased total reimbursement (Figure 1). The 32 patients in 2011 were reimbursed in total for less than half of that for the 34 patients in 2016. The average reimbursement for treatment per patient increased over the time frame, as shown in the trend line (Figure 2). There was wide variation, across and within years, and high outliers were common.

### 3.5. Quality of Life and Disability Adjusted Life Years

**Figure 3:**
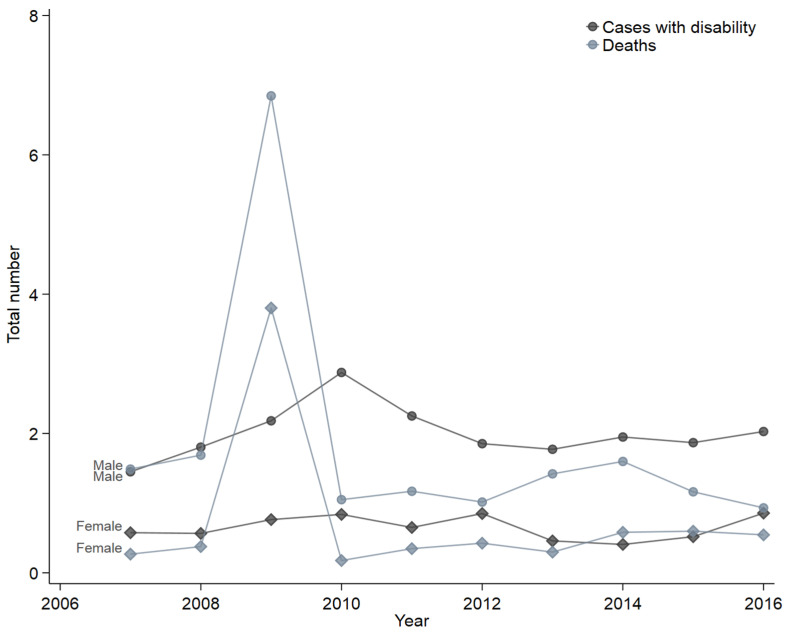
Cases with deaths and disability per 1,000,000 population by sex.

Disability weights at 6, 12, and 24 months were 0.156, 0.124, and 0.110 respectively. Of the 257 patients who survived to discharge, 55.8% were followed for two years, and 52.3% reported living with a disability (Table 3). Apart from 2009, when the burden of burn injuries was estimated to be 4737 DALYs, an average of 861 DALYs were lost each year due to burns. Excluding 2009, there were 7749 DALYs lost in total. They were costed at $991,872,000. The total for 2007–2016 was 12,486 DALYs at a cost of $1,598,208,000 [16]. Population incidence of cases with disability and deaths per 1,000,000 males and females is shown in Figure 3. More men died or lived with disability than women.

## 4. Discussion

The findings of this study provide an indication of the pattern and extent of costs that goes beyond previous burn costing studies, which primarily focus on acute health care costs over a relatively short time period [17]. Acute health care reimbursement costs are high but vary widely by patient and year. Despite severe injury, subsequent admissions in our cohort in the two years following the initial admission were uncommon, and most patients had no hospital readmissions during this period. This is lower than expected and possibly lower than reported in other services [18,19,20,21]. Health care use post-discharge is affected by social and psychological factors in addition to physical needs, indicating the need for active outreach if the specialist service is to provide optimal care [19,22]. Other possible explanations for the low rate of re-admission in the two years post injury include capacity constraints which necessitate prioritisation of acute patients, reluctance of patients to undergo further surgery soon after acute treatment, and low requirements of most patients for secondary surgery.

### 4.1. Health-Care Re-Imbursement

The average per patient total re-imbursement for acute treatment for our cohort with severe burns was $87,570 ($97,913). Costs were calculated for all patients, including those for whom no active treatment was commenced. A 2014 systematic review of the cost of burn care literature calculated mean total health costs per patient in high income countries of US$88,218 ($93,511), but highlighted a lack of consistency in methods and in reporting, which limits comparisons [17]. In 2008, an Australian patient-level costing study reported on a sample of 20 burns patients with a range of burn sizes. It determined that the average adult burns patient cost for acute treatment, including outpatient treatment, was $71,056 (US$73,532) [3]. This equates to $84,557 in 2016 and is only marginally less than the average reimbursement for our cohort, which included only severe burns. This study and others, along with our findings of large fluctuations in demand in a service with high fixed costs, highlights the recognised deficiencies of case mix in funding high-complexity low-volume services [7,23,24,25,26].

### 4.2. Overall Burden of Injury

Most DALYs were due to premature deaths, which included all burns-related deaths in Victoria. However, we identified significant ongoing levels of disability contributing to the burden of disease after burns. Our findings are broadly consistent with those of previous studies, including the Global Burden of Disease study, which calculated a long-term weight (CI) of 0.135 (0.092–0.190) [11] and others [27,28]. Studies indicate that while function in most domains recovers over time, disability in the domains of role participation, anxiety and depression and pain/discomfort persists [29]. In contrast, in their study of Finnish burns patients, Koljonen et al. related the costs of patient treatment to their health-related quality of life (HRQoL) as surveyed approximately two years post injury. Analysis of care costs and health-related quality of life scores did not show a consistent relationship. The respondents demonstrated a HRQoL that was similar to that for an aged and sex-standardised general population [30]. The authors concluded that despite high treatment costs, the outcomes justified the spending on burns treatment. Our study did not capture the costs of outpatient or community treatment, nor examine carer or lost working time costs associated with burn injury. Sanchez et al. [31] have to date provided the most comprehensive assessment of cost of burn injury. They estimated that direct health care costs accounted for only 19.6% of the total cost of a burn injury.

### 4.3. Limitations

This study examined hospital funding as determined by case-mix methodology. It did not have access to patient level costing data, nor specific details of funding allocated outside of the reimbursement described. For a small number of patients, information was insufficient to calculate re-imbursement, and average costs were assumed for those patients by the type of care and injury group. The rate of missing data was low and a complete case analysis was performed. Funding information for outpatient clinic care was not available. The DALY calculations are based on group average disability weights of available information (excluding data not available), which is a potential source of measurement error. Patients who died in hospital of burns <20%TBSA were not included in the study.

## 5. Conclusions

The costs of acute care and ongoing burden of injury caused by burns are high. Severe burns patients are expensive to treat, with fluctuating demand and costs by year. These findings, those from other centres, and the basic process for determining reimbursement in the Victorian case-mix system indicate that the actual cost of treatment is likely to be significantly underestimated using this method. Nonetheless, most direct clinical reimbursement costs are high and are incurred during the initial inpatient treatment. Without an understanding of the true cost of service provision, and a commitment to transparency and adequacy in funding allocation, expensive services such as burns are at risk of restriction of scope and will be hampered in providing innovation and research necessary for service development. Survivors have a low rate of re-presentation and a high ongoing burden of injury, with significant ongoing overall burden of injury associated with estimated lifetime health loss.

## Figures and Tables

**Table 1:** Patient and injury demographics.

Variable	% Total Body Surface Area			
	20–29%	30–39%	40–89%	All Patients
Total N	148	65	118	331
Demographics and injury characteristics				
Died	* Hidden, <10	9 (13.85%)	62 (52.54%)	74 (22.36%)
Male sex	114 (77.03%)	42 (64.62%)	90 (76.27%)	246 (74.32%)
Patient age category (years)				
16–34	57 (38.51%)	26 (40.00%)	29 (24.58%)	112 (33.84%)
35–64	67 (45.27%)	25 (38.46%)	70 (59.32%)	162 (48.94%)
65+	24 (16.22%)	14 (21.54%)	19 (16.10%)	57 (17.22%)
Length of stay (days), mean (SD)	26.71 (18.64)	36.58 (20.34)	37.23 (42.95)	32.40 (30.25)
ICU admission	69 (46.62%)	43 (66.15%)	89 (75.42%)	201 (60.73%)
Hours ventilated (If stayed in ICU), median (IQR)	40.00 (15.00, 194.00)	176.00 (57.00, 337.00)	193.00 (13.00, 498.00)	98.00 (16.00, 312.00)
Charlson Comorbidity Index (CCI)				
None	103 (69.59%)	36 (55.38%)	79 (66.95%)	218 (65.86%)
CCI = 1	29 (19.59%)	20 (30.77%)	13 (11.02%)	62 (18.73%)
CCI > 1	16 (10.81%)	9 (13.85%)	26 (22.03%)	51 (15.41%)
Intentional injury	17 (11.49%)	5 (7.69%)	43 (36.44%)	65 (19.64%)
Compensation status				
Not compensable	123 (83.11%)	56 (86.15%)	107 (90.68%)	286 (86.40%)
Compensable	25 (16.89%)	9 (13.85%)	11 (9.32%)	45 (13.60%)

* Exact value hidden to avoid possible statistical disclosure.

**Table 2:** Costs: initial injury and subsequent two years for all patients and by size of burn.

	20–29% TBSA	30–39% TBSA	40–89% TBSA	TOTAL
INITIAL INJURY TREATMENT				
Number of patients	148	65	118	331
Emergency Department (2016 AUD), median (IQR)	$1403 ($873, $1744)	$1364 ($873, $1691)	$1364 ($1250, $2145)	$1403 ($950, $1993)
Emergency Department (2016 AUD), mean (SD)	$1465 ($661)	$1454 ($591)	$1684 ($887)	$1542 ($745)
Hospital admission (2016 AUD), median (IQR)	$25,772 ($15,979, $83,028)	$93,173 ($26,120, $138,668)	$78,831 ($2,729, $214,092)	$37,256 ($12,398, $135,633)
Hospital admission (2016 AUD), mean (SD)	$52,867 ($57,980)	$102,902 ($91,202)	$118,401 ($124,823)	$86,055 ($97,769)
Total (2016 AUD), median (IQR)	$27,760 ($17,267, $84,476)	$94,405 ($27,523, $139,490)	$79,919 ($4,644, $214,981)	$39,343 ($13,844, $137,452)
Total (2016 AUD), mean (SD)	$54,292 ($58,177)	$104,312 ($91,357)	$120,085 ($124,899)	$87,570 ($97,913)
FOLLOW UP TREATMENT WITHIN 2 YEARS OF INJURY				
Number of ED visits, median (IQR)	0.00 (0.00, 1.00)	0.00 (0.00, 1.00)	0.00 (0.00, 0.00)	0.00 (0.00, 1.00)
Emergency Department (2016 AUD), median (IQR)	$730 ($375, $1518)	$829 ($380, $1307)	$1087 ($639, $1963)	$789 ($380, $1624)
Emergency Department (2016 AUD), mean (SD)	$1370 ($2022)	$1167 ($1113)	$1561 ($1466)	$1370 ($1757)
Number of Hospital admissions, median (IQR)	1.00 (0.00, 2.00)	0.00 (0.00, 1.00)	0.00 (0.00, 2.00)	0.00 (0.00, 2.00)
Hospital admission (2016 AUD), median (IQR)	$4648 ($2147, $10,985)	$5732 ($2198, $20,583)	$12,074 ($,5224, $25,659)	$6976 ($2303, $15,249)
Hospital admission (2016 AUD), mean (SD)	$9343 ($13,519)	$13,226 ($17,230)	$21,129 ($28,105)	$13,376 ($19,841)
Total (2016 AUD), median (IQR)	$2870 ($1057, $9788)	$ 3947 ($1307, $14,492)	$12,179 ($5160, $22,990)	$5120 ($1461, $13,699)
Total (2016 AUD), mean (SD)	$8172 ($13,275)	$ 11,163 ($16,756)	$ 20,936 ($28,492)	$11,853 ($19,320)

**Table 3:** DALYs per year.

Year	Cases with Disability	Total Deaths	Prehospital Deaths	Years of Life Lost (YLLs)	Years Lived with Disability (YLDs)	Disability Adjusted Life Years (DALYs)
2007	52	45	34	933	63	996
2008	62	54	41	1027	72	1099
2009	64	232	220	4665	72	4737
2010	82	27	21	538	102	640
2011	65	34	27	612	79	691
2012	62	33	29	732	73	805
2013	52	40	31	816	63	879
2014	56	52	41	962	66	1028
2015	58	43	31	778	66	844
2016	72	37	23	685	81	766

## Data Availability

Access to the data requires the relevant ethics and data custodian approvals.
